# The Socio-Demographic Characteristics Associated with Non-Communicable Diseases among the Adult Population of Dubai: Results from Dubai Household Survey 2019

**DOI:** 10.3390/healthcare9091139

**Published:** 2021-08-31

**Authors:** Wafa K. Alnakhi, Heba Mamdouh, Hamid Y. Hussain, Gamal M. Ibrahim, Amar Sabri Ahmad, Raghib Ali, Abdishakur Abdulle

**Affiliations:** 1 Department of Data Analysis, Research and Studies Department, Dubai Health Authority, Dubai 7272, United Arab Emirates; HMMohammed@dha.gov.ae (H.M.); hyHussain@dha.gov.ae (H.Y.H.); gmibrahim@dha.gov.ae (G.M.I.); 2 Mohammed Bin Rashid University of Medicine and Health Sciences, Dubai 505055, United Arab Emirates; 3 Department of Family Health, High Institute of Public Health, Alexandria University, Alexandria 21561, Egypt; 4 High Institute for Management Sciences, Belqas 35631, Egypt; 5 Public Health Research Center, New York University Abu Dhabi, Abu Dhabi 129188, United Arab Emirates; asa12@nyu.edu (A.S.A.); ra107@nyu.edu (R.A.); a.abdulle@nyu.edu (A.A.)

**Keywords:** non-communicable diseases (NCDs), sociodemographic characteristics, Dubai, complex survey design, multistage probability sampling, logistic regression model

## Abstract

Background: Non-communicable diseases (NCDs) are the leading causes of death worldwide. In the UAE, NCDs account for nearly 77% of all deaths. There is limited empirical research on this topic in the UAE. We aimed to examine the association of non-communicable diseases and the sociodemographic characteristics among the adult population of Dubai. Methods: The study used secondary data from the Dubai Household Health Survey (DHHS), 2019. DHHS is a cross-sectional complex design, stratified by geographic area, and uses multistage probability sampling. In this survey, 2247 families were interviewed and only adults aged 18+ were included for the analysis. The quasi-binomial distribution was used to identify the socio-demographic characteristics association with NCDs. Results: The prevalence of NCDs among the adult population of Dubai was 15.01%. Individuals aged 60+, local Arabs (Emirati), divorced and widowed individuals, and individuals who were not currently working reported NCDs more than the other groups. In the regression analysis, the association with NCDs were reported among elderly people, males, unmarried individuals, older individuals who are unmarried, and Emiratis. Conclusion: The study identified several socio-demographic characteristics associated with reporting NCDs. This is one of the few studies related to NCDs in Dubai. Allocating appropriate resources to the population groups identified is crucial to reduce the incidence of NCDs in the Emirate.

## 1. Introduction

Non-communicable diseases (NCDs) are the leading cause of death worldwide. Based on the WHO projections, NCDs will account for over 70% of all deaths globally by 2025, with 85% of these occurring in developing countries [1]. In addition, one in three adults lives with one or more chronic conditions worldwide [2]. In the United Arab Emirates (UAE), NCDs account for nearly 77% of all deaths, putting increasing strain on the well-being of the population, economic development, and the healthcare system [3].

Data from the most recent UAE population-based survey revealed that 13.7% of the population reported having diabetes and 17.8% reported having hypertension [4]. Similar results were reported in surveys conducted in Kuwait, Oman and other neighboring Gulf States [5,6,7]. In Kuwait, for example, the most recent population survey showed a high burden of self-reported NCDs, with 11.8% and 17.7% of the respondents had diabetes and hypertension [5].

Dubai is one of the fastest-growing emirates in the UAE, with its unique multiethnic, young, and male-dominated population structure [8]. The change in socioeconomic status in Dubai has resulted in significant alterations in the living conditions as well as the health and illness patterns of the population [9]. Traditional eating habits have shifted, and a sedentary and technology-driven lifestyle has become ingrained in the culture, leading to a growing burden of NCDs [10]. As per the Emirate of Dubai law, it is mandatory that all residents have minimum health insurance coverage [11]. In addition, all Emirati citizens, irrespective of sociodemographic characteristics, are eligible for quality healthcare services. Therefore, it is important to understand the factors associated with of NCDs to better allocate healthcare services and resources to individuals or groups who might be at higher risk among the Dubai population [12].

The causes of NCDs are multifactorial; these diseases may arise from a combination of underlying, modifiable and non-modifiable risk factors [13]. Research indicates that non-modifiable risk factors for NCDs include age, genetic predisposition, and ethnicity [14]. The presence of NCDs and many of their risk factors have been found to be more common in population groups with low income and low educational attainment in both low-middle income and high-income countries [15,16]. The relationship between sociodemographic characteristics and chronic conditions differs largely by geographic region [17]. Identifying the priority population groups that are the most affected by the growing burden of NCDs has become necessary to target appropriate health promotion and prevention programs.

The government of Dubai continues to regularly monitor population health by conducting the Dubai Household Health Surveys (DHHS). In general, self-reported data are widely used in social science and epidemiological research [18,19]. Although it is preferable to have measures of disease from medical records or objective measures, useful data on disease status can be collected when interviewees report their disease burden [20,21]. Since self-reported data can provide a useful estimate for broad measures of population rates, this study has used self-reported data from DHHS 2019 to estimate the NCDs among the population of Dubai. To our knowledge, no studies investigated the effects of sociodemographic characteristics on NCDs in the Emirate of Dubai. Therefore, this study aimed to contribute to regional and global literature by examining the association between sociodemographic characteristics and NCDs as reported by a representative sample of the Dubai population.

## 2. Materials and Methods

### 2.1. Data Source, Study Design

The data were obtained from DHHS 2019, which was carried out between February and March 2019 as a collaborative work between the Dubai Health Authority (DHA) and Dubai Statistics Center (DSC). The DHHS is a series of representative population surveys that have been carried out in 5-year intervals since 2009. The survey covers a wide range of health issues related to mortality, health expenditures, access to health services, health-related behaviors and other topics related to population health of Dubai. The DHHS covered socio-demographic characteristics, economic aspects, and behavioral risk factors. The DHHS is a cross-sectional complex study design, stratified by geographic area, and uses multistage probability sampling. The methods used in the survey are described in detail elsewhere [22]. The design and methodology of the survey were adopted from World Bank’s Living Standards Measurement Surveys, World Health Organization’s World Health Survey (WHS) and the U.S. Centers for Disease Control’s National Health Interview Surveys (NHIS). Of a total of 2247 families, 9630 individuals were interviewed with a response rate of 91.6%. The sampling frame of the survey is all residents of Dubai. Each sector in Dubai is divided into areas, and each area is divided into blocks that are given a specific numerical identifier. The target population of this survey was Emirati and non-Emirati families. Sampling weights were used for this study to account for the complex survey design. Only adults aged 18+ were included in the final analysis (75.35%).

### 2.2. Variables and Measures

Sociodemographic variables were reported in weighted percentages: birth date, gender, marital status, nationality, educational attainment, and working status. Birth date was converted to four age groups as categorical and continuous variables. Gender was dichotomized to male and female. Marital status was categorized as married, unmarried, or others (divorced and widowed). Nationality was a categorical variable with 4 groups: Emirati, Asians (All Asian nationalities), other Arabs (Any Arab nationalities that consists of 22 countries in the Middle East and North Africa other than Emirati (nationals)), and other nationalities (Any other nationalities not mentioned in the above categories). Educational attainment was a categorical variable with 3 groups and defined as less than secondary education, completed secondary school and tertiary education ( Postsecondary education (beyond high school, including undergraduate and graduate credentials)). Throughout this manuscript, NCDs are defined as self-reported, existing, chronic conditions from a predefined list as per the WHO criteria for the most common NCDs [23]. Participants were asked if they ever had been diagnosed with one or more of the following NCDs: diabetes mellitus, high blood pressure, asthma, cancer, arthritis and mental or physical disabilities. Complete case analysis was considered in this study, and 17 missing NCD responses were excluded from the statistical analysis.

### 2.3. Statistical Analysis

Categorical variables are reported in weighted frequencies and percentages, which were normalized to the total of the primary sampling unit. Chi-square tests for association in the survey data were used to investigate the association between NCDs (combined) and the independent variables [24]. Design-based versions of the Wilcoxon test and Kruskal-Wallis test were performed as appropriate. A multivariate logistic regression model for complex survey design was performed to investigate the influence of sociodemographic variables on NCDs. The self-reported NCDs variable was used as a binary outcome. The quasi-binomial distribution was used to correct for over and under-dispersion. The predictors were age, nationality, marital status, gender, educational attainment, and work status. To evaluate the effect of age and marital status on self-reported NCD, an interaction term between age and marital status was added to the multivariate model and tested using the Rao-Scott test. The model with and without the interaction term showed that the interaction term adds a significant amount of information to the final multivariate model (*p*-value = 0.013). To compute interquartile range odds ratios (ORs) for age (years), age was divided by its interquartile range before it was added to the logistic regression models, and 95% confidence intervals (95% CIs) were estimated [25]. All applied statistical tests were two-sided, and *p*-values < 0.05 were considered significant. No adjustment for multiple comparisons was made. Statistical analyses were performed in R version 3.6.1 [26].

## 3. Results

### 3.1. Sociodemographic Characteristics of the Population of Dubai

**Figure 1:**
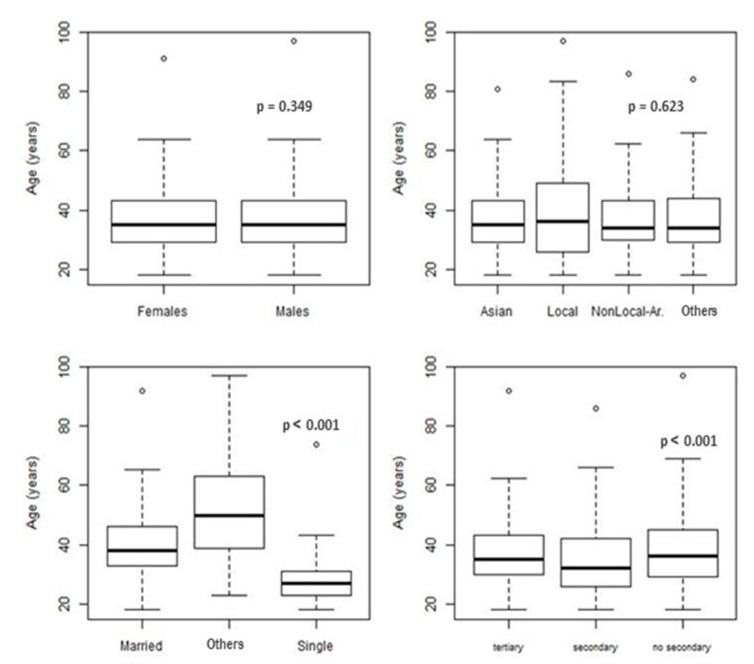
Distribution of Age by Gender, Nationality, Marital Status and Education Groups.

Overall, 15.01% of the surveyed population self-reported having at least one of the NCDs on the predefined list in the survey during the past year. Figure 1 shows the distribution of age by gender, nationality, marital status, and education group. Significant differences were observed among marital status, since unmarried individuals were younger than those who were married and others (divorced and widowed), *p*-value < 0.001. Furthermore, significant differences were observed between the age distributions by education group, *p*-value < 0.001. Table 1 illustrates the frequency distribution of sociodemographic characteristics among the Dubai population (aged 18+). Among the age groups, individuals aged 60+ were most likely to report having NCDs (48.03%), *p*-value < 0.001. Local Arabs (Emirati) reported the highest frequency of NCDs (25.14%), *p*-value < 0.001. Divorced and widowed individuals were most likely to report NCDs (34.21%), *p*-value < 0.001. Individuals who were not currently working reported NCDs more often than those who were working currently (17.72% versus 11.39%, respectively), *p*-value < 0.001. Males and less-educated individuals reported a slightly higher frequency of NCDs, but the difference was not significant. No statistically significant differences were observed in the income distribution (*p*-value = 0.161).

### 3.2. Associations with Non-Communicable Diseases

Table 2 shows the results of the multivariate logistic regression model used to examine the factors associated with reporting of NCD. After adjusting for age, gender, marital status, and educational attainment, age (years) was the strongest predictor of NCD in the multivariate logistic regression IQR-OR 3.6 (95% CI: 2.8, 4.7). Unmarried individuals reported fewer NCDs than married individuals (OR 0.11, 95% CI: 0.02, 0.70). However, older individuals who were unmarried were more likely to report NCD compared to the reference group of older individuals who were married (OR 2.8, 95% CI: 1.2, 6.1). Emiratis were more likely to report NCDs than the reference group, Asians (OR 2.08, 95% CI: 1.62, 2.67). Males were more likely to report NCDs than females (OR 1.61, 95% CI: 1.277, 2.035).

## 4. Discussion

Our study is the first to report on the estimates of self-reported NCDs among the adult population of Dubai. The results reveal a high prevalence of self-reported NCDs (combined) among the adult population of the Emirate of Dubai, UAE. Furthermore, our results reveal that a number of non-modifiable risk factors are associated with NCDs in this population. The high crude prevalence (15%) of self-reported NCDs found in this survey is in line with what has been reported previously across the Arabian Gulf states [7,27,28,29]. Because of logistical challenges, objective measures of NCDs did not provide adequate data for the current analysis. However, population-based self-reporting of NCDs has been shown to be relatively reliable [30]. Although non-communicable diseases are caused by a combination of modifiable and non-modifiable risk factors [31], our analysis was limited to non-modifiable risk factors. These characteristics are essential to understand the context, profile, and trend of non-communicable diseases among the population. Of the variables analyzed, age, gender, marital status, and nationality were found to be associated with self-reported NCDs as defined in our study methods.

While Dubai is a major metropolitan city serving as a melting pot of diverse nationalities [9], Emiratis were found to report a higher rate of NCDs than other nationalities in the Emirate. Interestingly, though not surprisingly, an association was found between NCDs and older individuals who remained unmarried. Previous studies reported associations between sociodemographic characteristics and NCDs [15,29,32,33]. Our results confirmed that the prevalence of chronic conditions increases with age in concordance with previously published studies in the UAE [33,34,35]. Other studies in the region have revealed similar findings related to the association of age with non-communicable diseases [27,28,29,36]. Obviously, the cumulative effect of NCDs over time and the degenerative process combined with aging contribute to the development of different types of NCDs. This is especially important considering Dubai’s unique age structure, with 65% of its population being between 25–48 years old, which signals that once this age group ages, the prevalence of NCDs is expected to increase in the Emirate.

Our results revealed that nationality is one of the non-modifiable risk factors and a predictor of the report of NCDs. This finding has been echoed in many studies, and sometimes nationality was referred to as ethnicity [37,38,39,40]. In this analysis, we found that Emiratis had a higher likelihood of reporting NCDs than other nationalities when adjusting for other sociodemographic characteristics. Another study that examined diabetes and impaired fasting glycemia in the UAE revealed that these conditions were higher in Emiratis than in other nationalities [39]. Similarly, for cardiovascular diseases, a study has found that these morbidities are more prevalent in some nationalities and ethnicities than others [33]. These findings could be due to lifestyle, genetics, cultural and/or socioeconomic factors as explained in many previous publications [38,41].

Gender was found to be another significant predictor for NCDs among the residents of Dubai. Different studies have examined the gender difference in NCDs and the experience of these illnesses related to gender identity [41,42,43,44,45]. Men are more prone to a wider range of health risks, such as hypertension, cerebrovascular diseases, and cardiovascular diseases [45]. On the other hand, women are more likely to report diseases related to arthritis, osteoporosis diabetes and hypertension [46]. Moreover, since men tend to adopt unhealthy behaviors, such as smoking and alcohol consumption, they are more likely to report experiencing NCDs and poor health than females because of the negative consequences of such behavioral lifestyle on health outcomes [41,45].

In the current study, unmarried adults were reported to have a lower risk of NCDs than married and formerly married adults. However, when combining age with marital status, the relationship was reversed, and NCD was higher in older unmarried individuals within our population sample. Unmarried individuals who were older had a three-fold risk of reporting NCDs compared to older married individuals. Although published data show that, the association between NCDs and marital status is scarce and old [47,48,49], there are many published results demonstrating higher risk of mortality among unmarried individuals [50,51,52]. Moreover, a longitudinal study in the United States identified that marital status was associated with poor health outcomes at the oldest ages [53]. This association can be attributed to the selective effect of marriage, where marriage can sometimes reduce stress and provide social support in light of clarity in the defined roles between married couples.

It is important to acknowledge some limitations of our study. The data on presence of NCDs for this study was derived from individual self-reports and not from medical records. Although self-reporting is a widely used practice to reflect patient experience, it may include subjectivity as a diagnostic tool with bias and accuracy. Self-reporting might lead to under- or over reporting on some occasions. Another limitation involved in this study, the severity of the NCD and the existence of comorbidity were not included, and these qualifiers may better explain the status of individuals.

Due to under reporting of behavioral risk factors in our study, our analysis was limited only to the non-modifiable sociodemographic characteristics. Under reporting of smoking and alcohol consumption is very common in conservative cultures such as the Arab region, especially among women, since it might be linked to social stigma [54,55,56,57,58]. While the DHHS was limited to the population of Dubai, we cannot generalize the results to the general population of the UAE. However, the availability of the data can be used as a strength, especially because the survey tool is valid and was conducted through well-trained surveyors. Additionally, the data can be used in the future to conduct trend and pattern analyses for monitoring Dubai population health.

## 5. Conclusions

In conclusion, the results of this study, with a substantially large sample size, provide important NCD data for the population of Dubai and indeed contribute to limited empirical research in the emirate of Dubai and the UAE in general. Our results demonstrated that older age, unmarried status, male, and Emirati nationality are significant predictors of NCDs. Understanding the dynamics of age, ethnicity, and gender and their association with NCDs can help inform health interventions and priorities. The current findings could be translated to policies and strategies to reallocate healthcare services to create more effective health prevention and promotion programs aligned with government of Dubai Health agenda. Therefore, the DHA and the Government of Dubai are advised to enforce policies aimed at alleviating the risks of NCDs among its residents in light of the forecasted increase in NCDs prevalence when the current young demographic of Dubai ages. Such policies may include more resources for primary healthcare infrastructure and support for behavioral and lifestyle changes (e.g., tobacco cessation, healthy diets, physical activity, etc.). This process includes improving the quality of current programs or initiating new programs related to the targeted population in our study. In the future, it is important to conduct in-depth assessments of patients’ quality of life and experience with chronic conditions to reduce the complications associated with NCDs. Besides, it is recommended to conduct further prospective studies to look at each NCD separately and its association with the socio-demographic characteristics.

## Figures and Tables

**Table 1:** Sociodemographic characteristics of the Dubai population with self-reported non-communicable diseases from the DHHS 2019.

Variable	Category	Having NCDs		Wald’s χ_1_^2^ Test (ndf)	*p* Value
		No	Yes		
Age groups (years)	18–24	298,774 (95.86%)	12,893 (4.14%)	39.03 (3)	<0.0001
	25–44	1,644,723 (92.57%)	132,052 (7.43%)		
	45–59	344,062 (71.86%)	134,735 (28.14%)		
	60+	54,435 (51.97%)	50,300 (48.03%)		
Gender	Female	697,618 (89.29%)	83,644 (10.71%)	3.39 (1)	0.067
	Male	1,644,376 (86.97%)	246,336 (13.03%)		
Nationality	Asians	1,629,905 (87.85%)	225,415 (12.15%)	37.17 (3)	<0.0001
	Local-Arabs (Emirati)	117,901(74.68%)	39,604 (25.14%)		
	Non-Local-Arabs	253,466 (90.25%)	27,373 (9.75%)		
	Other nationalities	340,722 (90.16%)	37,589 (9.94%)		
Marital Status	Married	1,551,962 (85.25%)	268,558 (14.75%)	32.42 (2)	<0.0001
	Single	751,578 (94.78%)	41,425 (5.22%)		
	Others *	38,454 (65.79%)	19,997 (34.21%)		
Work Status	Currently working	2,008,951 (88.61%)	258,271 (11.39%)	18.33 (1)	<0.0001
	Currently not working	333,043 (82.28%)	71,709 (17.72%)		
Educational Attainment	Less than secondary education	570,458 (85.13%)	99,666 (14.87%)	1.31 (2)	0.271
	Completed secondary school	525,871 (88.27%)	69,888 (11.73%)		
	Tertiary education	1,245,666 (88.59%)	160,427 (11.41%)		
Income Categories	Low Income	344,101(88.78%)	43,509 (11.22%)	1.74 (3)	0.161
	Middle income	188,026 (85.96%)	30,714 (14.04%)		
	High income	209,398 (83.76%)	40,611 (16.24%)		
Total		84.99%	15.01%		

Wald’s χ_1_^2^ test corresponding numerator degrees of freedom (ndf) are listed in the table, while the denominator degrees of freedom was 201. * Others refers to formerly married or widowed and divorced individuals.

**Table 2:** Multivariate logistic regression model for NCD among the Dubai Population from DHHS 2019.

Variable	Category	Odds Ratio (95% CI)	Wald’s χ_1_^2^ Test	*p* Value
Age (years)	18–60+	3.6 (2.8, 4.7)	90.32	<0.001
Marital Status	Married	(Reference)		
	Unmarried	0.11 (0.02, 0.70)	5.43	0.020
	Others	0.32 (0.04, 2.32)	1.28	0.258
Nationality	Asians	(Reference)		
	Local-Arab (Emirati)	2.08 (1.62, 2.67)	32.49	<0.001
	Non-Local (other Arabs)	0.72 (0.45, 1.15)	1.94	0.163
	Other Nationalities	0.81 (0.56, 1.18)	1.19	0.275
Gender	Female	(Reference)		
	Male	1.61 (1.28, 2.04)	16.107	<0.001
Educational Attainment	Education tertiary education	(Reference)		
	Education less than secondary school	1.09 (0.73, 1.64)	0.181	0.671
	Completed secondary school	1.18 (0.80, 1.74)	0.69	0.407
Age and marital Status	Age & married marital status	(Reference)		
	Age & unmarried marital status	2.75 (1.24, 6.09)	6.17	0.013
	Age & other marital status	1.56 (0.86, 2.85)	2.12	0.146

Estimated odds ratios with 95% confidence intervals and Wald’s chi-square tests with one degree of freedom and their corresponding *p*-values.

## Data Availability

The data that support the findings of this study are from the Dubai Health Authority. However, restrictions apply to the availability of these data; thus, these data are not publicly available. Data are available from the corresponding author upon responsible request with permission from the Dubai Health Authority.

## Abbreviations

DHA: Dubai Health Authority, DHHS: The Dubai Household Health Survey, DSC: Dubai Statistics Center, WHO: World Health Organization, NCDs: Non-Communicable Diseases.
